# Ozone treatment improved the extractability, bioaccessibility, and hypoglycemic potential of fresh-cut pitaya polyphenols

**DOI:** 10.3389/fnut.2025.1707700

**Published:** 2025-11-27

**Authors:** Chen Li, Yishuang Hu, Jinmiao Fang, Qiannuo Du, Han Ding

**Affiliations:** 1 College of Food and Health, Jinzhou Medical University, China; 2 Liaoning Provincial Professional Technology Innovation Center of Meat Processing and Quality-Safety Control, China

**Keywords:** fresh-cut pitaya, ozone, phenolic compounds, bioaccessibility, α-glucosidase, α-amylase

## Abstract

Fresh-cut fruits suffer from quality deterioration and low bioaccessibility of bound polyphenols, which limit their health benefits. This study used ozone treatment (10 μL/L for 60 min) to enhance the functional properties of fresh-cut pitaya. This treatment significantly (*p* < 0.05) disrupted the cell wall microstructure, increasing the extractability of free and bound phenols by 16.7 and 4.9%, respectively. This structural modification enhanced polyphenol bioaccessibility by 8% after *in vitro* digestion. Furthermore, the ozone-treated samples exhibited stronger inhibition of α-amylase and α-glucosidase, and *in silico* analyses suggested that the released polyphenols (e.g., naringin and ferulic acid) could modulate glucose metabolism via the PI3K-AKT pathway. The main advantage of this study is the direct linkage of ozone-induced microstructural changes to improved physiological functionality, positioning mild ozone treatment as a highly promising non-thermal technology that can produce health-enhanced fresh-cut fruits for the functional food market.

## 1 Introduction

Pitaya (dragon fruit), renowned for its vibrant color and rich nutrient profile, has gained global popularity as a functional food (1). Beyond its macronutrients, pitaya is a valuable source of bioactive compounds, particularly polyphenols, which are associated with potent antioxidant and antidiabetic activities (2). The growing consumer demand for convenient and healthy options has propelled the market for fresh-cut fruits, with pitaya being a prominent candidate owing to its appealing flesh and mild taste.

However, the fresh-cut processing industry faces several challenges. Mechanical operations such as cutting and peeling induce tissue damage and accelerate enzymatic browning, microbial spoilage, and quality deterioration (3, 4). From a nutritional standpoint, a substantial portion of polyphenols in plant foods exists in bound forms complexed with cell wall structures, such as cellulose and hemicellulose. This structural entrapment severely limits their release during human digestion, resulting in low bioaccessibility (the fraction released from the food matrix and available for intestinal absorption) and consequently diminished health benefits (5). Therefore, developing processing technologies that can preserve quality and enhance bioaccessibility of bound polyphenols is a key objective for the functional food sector.

Ozone (O₃) treatment has emerged as a promising green technology for postharvest management. Ozone, with its Generally Recognized as Safe (GRAS) designation, is primarily employed for its strong antimicrobial efficacy, which can effectively decontaminate fruit surfaces and extend their shelf life (6, 7). Beyond this conventional role, emerging evidence suggests that ozone also acts as a physical elicitor in plant tissues. Its oxidative properties may induce mild stress responses and, more importantly, modify the structural integrity of the cell wall matrix by breaking down polysaccharides (8). This modification presents a compelling opportunity to enhance the extractability of bound phytochemicals. Although recent studies on other crops have suggested this potential, a clear mechanistic link between ozone-induced microstructural changes, subsequent polyphenol bioaccessibility, and specific health-related functionalities in fresh-cut pitaya remains largely unexplored (7, 9, 10). Specifically, there is a notable research gap in understanding whether structural modifications caused by ozone translate into improved hypoglycemic potential—a critical health benefit of polyphenols—through enhanced bioaccessibility. However, the interplay between these factors has not been systematically investigated.

Therefore, the objectives of this study were to (1) evaluate the impact of ozone treatment on the microstructure of fresh-cut pitaya; (2) determine its effect on the extractability and *in vitro* bioaccessibility of free and bound polyphenols; (3) assess the subsequent changes in the *in vitro* hypoglycemic activity (α-amylase and α-glucosidase inhibition); and (4) explore the potential mechanisms of action through integrated *in vitro* and *in silico* analyses. This study aimed to establish ozone treatment not only as a preservation tool but also as a novel strategy to amplify the health-promoting value of fresh-cut pitaya.

## 2 Materials and methods

### 2.1 Plant material

Red pitaya fruit was sourced from its origin, following the procedure described in our previous study (11). Fruits of uniform maturity, size, color, and shape were selected with no signs of insect infestation or mechanical damage. The peel was manually removed, and the fruit was cut into quarters (approximately 1 cm thick). The segments were randomly distributed in polypropylene containers (15 × 10 × 4 cm), with each container containing approximately 150 g of sample. The samples were subsequently subjected to ozone fumigation and stored at 8 ± 2 °C for up to 72 h. During the storage period, samples were collected at regular intervals, immediately frozen in liquid nitrogen, and stored at −80 °C until further analysis.

### 2.2 Ozone treatment

Ozone was generated using a corona-discharge ozone generator (12). Dry oxygen was introduced into the corona discharge tube to produce pure gaseous ozone. Ozone gas was delivered to a sealed treatment chamber equipped with an inlet, outlet, and ozone concentration detector. The ozone concentration in the chamber was regulated by adjusting the flow rate of the ozone generator and monitored in real time using a gas detector. Fresh-cut pitaya samples were fumigated with ozone at a concentration of 10 μL·L^−1^ for 60 min. Fresh-cut pitaya fruit that did not receive ozone treatment served as the control group.

### 2.3 Scanning electron microscopy (SEM) and Fourier transform infrared (FTIR) spectroscopy

The samples were designated as follows: control group on day 0 (CKD0, fresh-cut pitaya without ozone treatment) and treatment group on day 0 (TD0, fresh-cut pitaya with ozone treatment); the control and treated samples after 2 days of storage were labeled as CKD2 and TD2, respectively.

For SEM analysis, the samples were mounted on a specimen stub using adhesive tape and were sputter-coated with a conductive layer. Observations were performed using a field-emission scanning electron microscope (Hitachi S4800, Japan) at an accelerating voltage of 2.00 kV. Images were captured at a magnification of 500 × under high-vacuum conditions (13).

FTIR analysis was performed using an FTIR spectrometer (Nicolet 6,700; Thermo Fisher Scientific, United States). The powdered sample was mixed with potassium bromide (KBr) at a ratio of 1:100 (w/w) and pressed into a transparent pellet. Spectra were acquired in the range of 4,000–400 cm^−1^ with 32 scans at a resolution of 4 cm^−1^. Peak identification and spectral processing were performed using the instrument software (13).

### 2.4 Effects of ozone treatment on phenolic content and antioxidant capacity of fresh-cut pitaya fruit

#### 2.4.1 Polyphenol extraction

Free phenolic compounds were extracted using a slightly modified version of the method described by Ding and Morozova et al. (5). Briefly, 1 g of each sample was homogenized with 10 mL of methanol and magnetically stirred for 40 min. The mixture was centrifuged at 4,000 × *g* for 10 min and the supernatant was collected. The residue was re-extracted twice under the same conditions. The combined supernatants were transferred into a brown reagent bottle and stored at 4 °C until further analysis.

The bound phenolic compounds were extracted from the remaining residue according to a modified protocol described by Ding et al. (14). The residue was hydrolyzed with 20 mL of 4 mol/L NaOH solution in the dark for 24 h. The pH of the mixture was then adjusted to 7.0 using concentrated hydrochloric acid, followed by centrifugation at 4,000 × *g* for 10 min. The supernatant was mixed with three volumes of 95% ethanol and stored at 4 °C for 12 h to precipitate polysaccharides and proteins. After centrifugation under the same conditions, the resulting supernatant was collected and stored in a brown bottle at 4 °C for subsequent use.

#### 2.4.2 Determination of polyphenol content

The total phenol content was determined using the method described by Gutiérrez and Chaves et al. (9), which is consistent with the experimental method described in our previous article (11).

#### 2.4.3 1,1-Diphenyl-2-picrylhydrazyl (DPPH) method

The antioxidant activities of the samples were evaluated according to the methodology described by Li et al. (3). The absorbance was measured at 515 nm to calculate the antioxidant activity as follows (Equation 1):


(1) $$\text{Antioxidant activity}\;(\%)=[(A_{0}-A_{1})/A_{0}]\times 100$$

where A_0_ and A_1_ are the absorbance values of the samples from the CK and ozone groups, respectively.

#### 2.4.4 Ferric-reducing antioxidant power (FRAP) assay

The total antioxidant capacity of the samples was measured using the ferric-reducing antioxidant power analysis, as previously described by Fu and Xu et al. (15). The results were expressed as mmol Fe^2+^ kg^−1^.

### 2.5 Determination of *α*-glucosidase and α-amylase inhibition rate

Fresh-cut pitaya polyphenols in the control group on day 0 (CKD0) and day 2 (CKD2) and fresh-cut pitaya polyphenols with ozone treatment on day 0 (TD0) and day 2 (TD2) were evaporated by rotation at 40 °C to remove the solvent. The vacuum was then freeze-dried into a powder and set aside. According to the method described by Liu et al. (16), different concentrations of pitaya polyphenol solutions were used to study the inhibition of *α*-glucosidase and α-amylase activities. Acarbose was used as a positive control. The experiment was conducted in triplicate. The formula used to calculate the inhibition rate is as follows (Equation 2):


(2) $$\begin{array}{l} \text{Inhibition rate of enzyme activity}\;(\%)\\ =[1-(A_{\text{sample}}-A_{\text{control}})/A_{\text{blank}}]\times 100\;\; \end{array}$$

where A_sample_, A_control_, and A_blank_ are the absorbances of the sample, control, and blank, respectively.

The *α*-glucosidase and α-amylase inhibition effects were expressed as half-maximal inhibitory concentration (IC_50_) values, represented as mg/mL.

### 2.6 *In vitro* simulation of gastrointestinal digestion

Fresh-cut pitaya fruit (20 g) was extracted according to the method described in Section 2.4.1, and freeze-dried pitaya powder, free phenols, and combined phenol samples were prepared. The method of Pea-Vázquez and Dominguez-Fernández et al. (17) was used to simulate saliva, gastric fluid, and intestinal fluid. After digestion, the samples were immediately frozen at −18 °C to terminate enzymatic activity and subsequently stored at −80 °C until lyophilization. The total phenolic content of the digested samples was determined using the method outlined in Section 2.4.2. The bioaccessibility of polyphenols was calculated as the percentage of total phenolic content (TPC) in the bioaccessible fraction (the supernatant after digestion and centrifugation) relative to the TPC in the undigested sample. All calculations were based on the fresh weight of the pitaya pulp.

### 2.7 *In silico* evaluation of the hypoglycemic potential of fresh-cut pitaya polyphenols

#### 2.7.1 Selection of active ingredients and pharmacokinetic evaluation

Based on our previous study, which identified 26 phenolic compounds in red pitaya fruits (11), these compounds were submitted to the SWISSADME database^1^ for pharmacokinetic profiling. A bioavailability score greater than 0.55 was set as the main criterion for selecting compounds with potential biological activities (18). Seventeen compounds met this criterion and were selected for further analyses (Table 1).

**Table 1:** Active components of fresh-cut pitaya polyphenols.

Compound name	Formula	Structure	Bioavailability score
trans-Cinnamic acid	C_9_H_8_O_2_	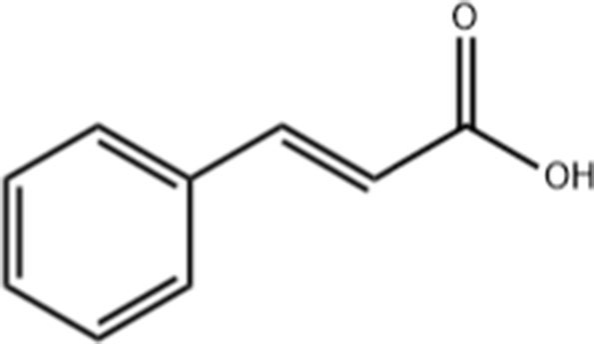	0.85
Vanillic acid	C_8_H_8_O_4_	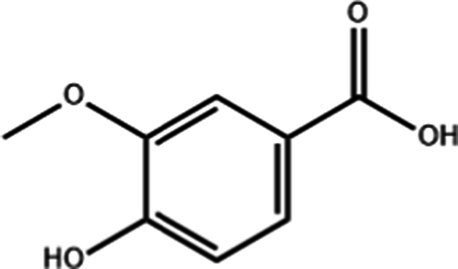	0.85
4-Hydroxybenzoic acid	C_7_H_6_O_3_	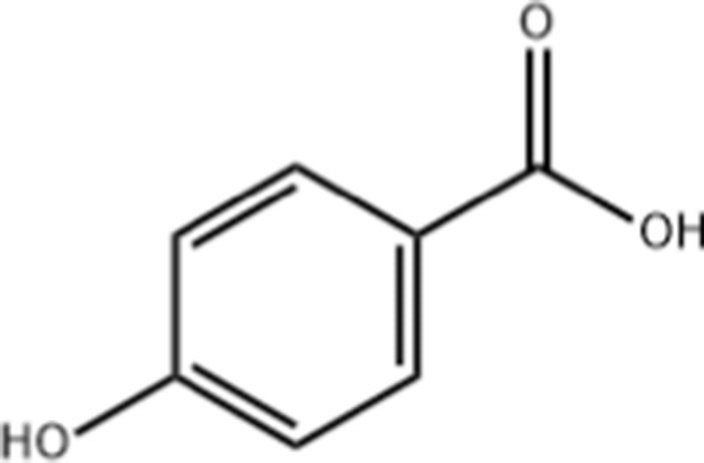	0.85
Salicylic acid	C_7_H_6_O_3_	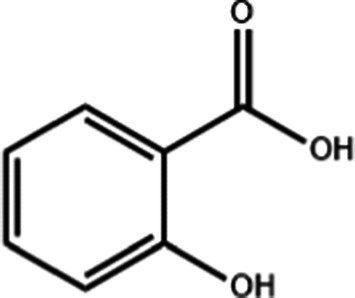	0.85
4-Hydroxycinnamic acid	C_9_H_8_O_3_	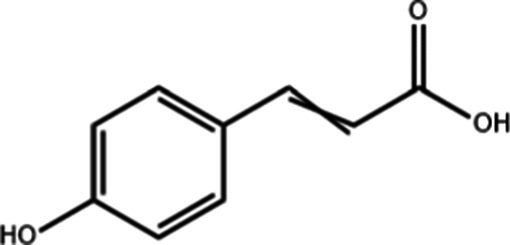	0.85
Ferulic acid	C_10_H_10_O_4_	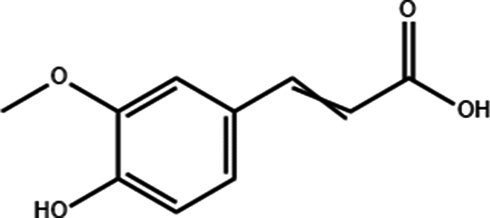	0.85
Sinapic acid	C_11_H_12_O_5_	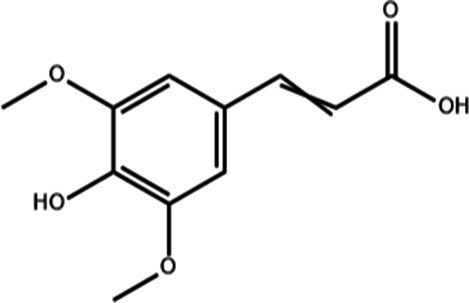	0.85
Protocatechuic acid	C_7_H_6_O_4_	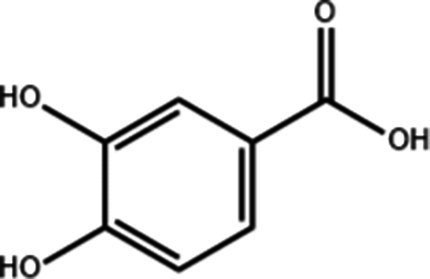	0.56
Caffeic acid	C_9_H_8_O_4_	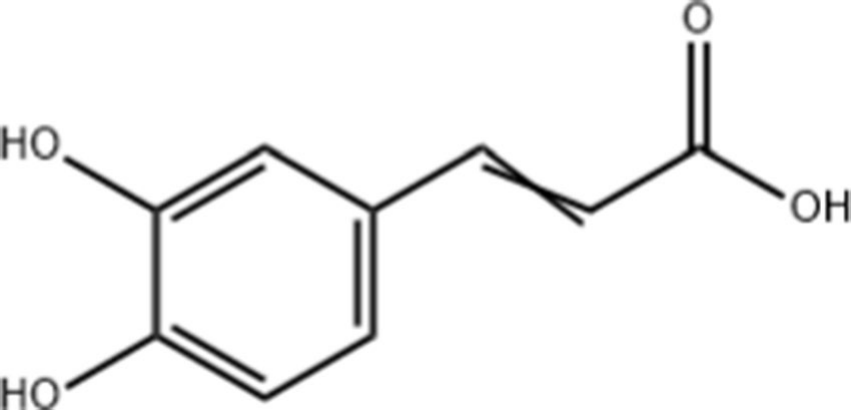	0.56
3,4-Dihydroxybenzaldehyde	C_7_H_6_O_3_	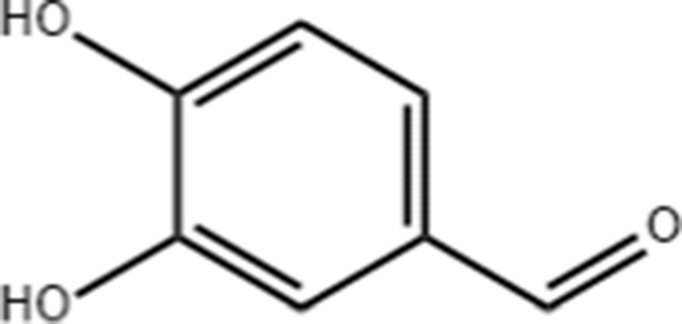	0.55
Catechin	C_15_H_14_O_6_	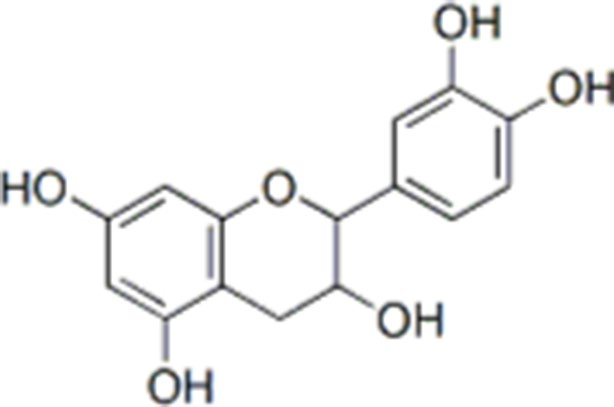	0.55
Epicatechin	C_15_H_14_O_6_	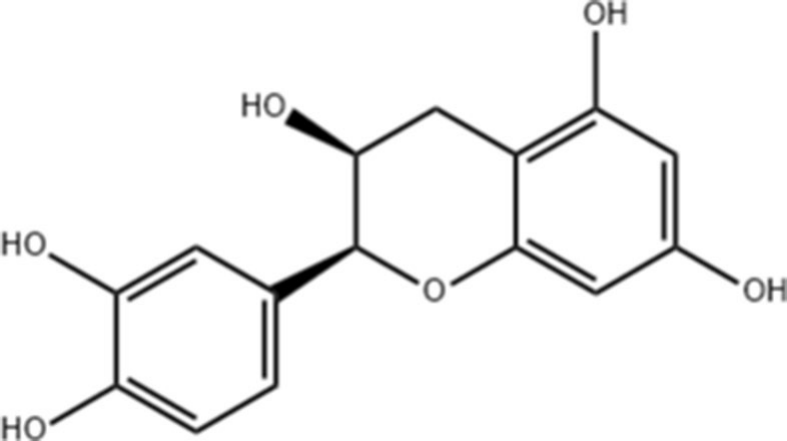	0.55
Syringaldehyde	C_9_H_10_O_4_	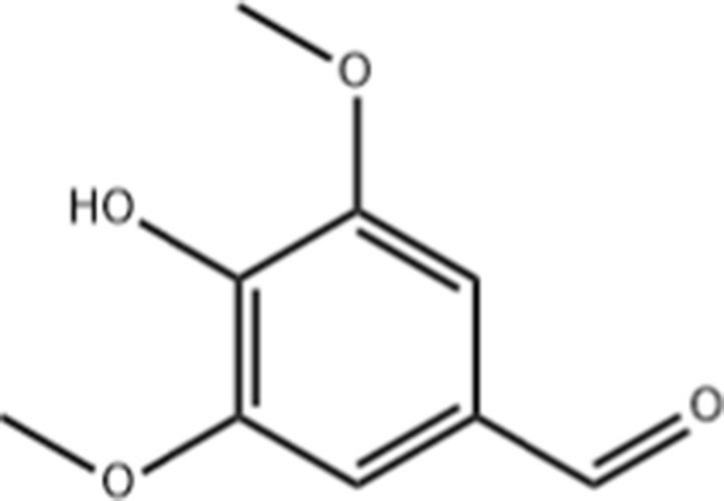	0.55
Salicin	C_13_H_18_O_7_	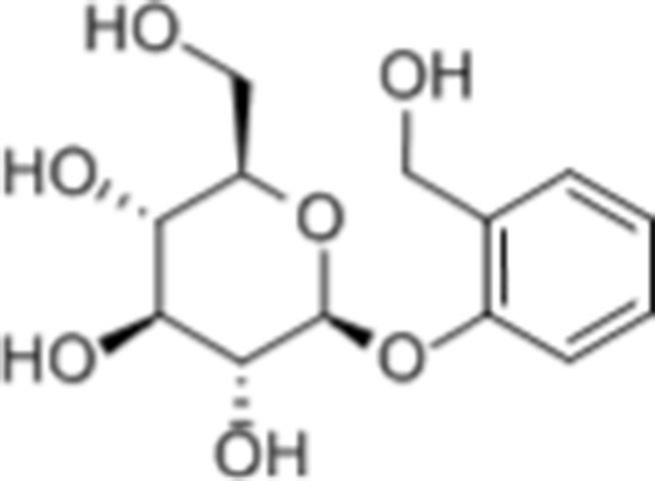	0.55
Naringenin	C_15_H_12_O_5_	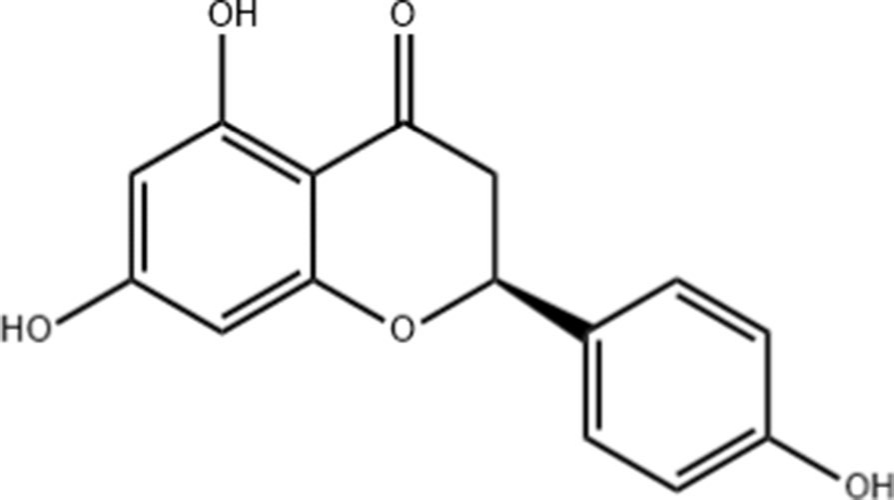	0.55
Aesculin	C_15_H_16_O_9_	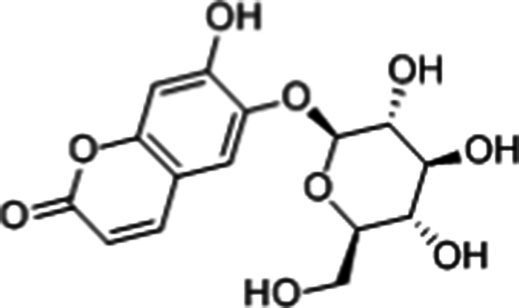	0.55
4-Methylumbelliferone	C_10_H_8_O_3_	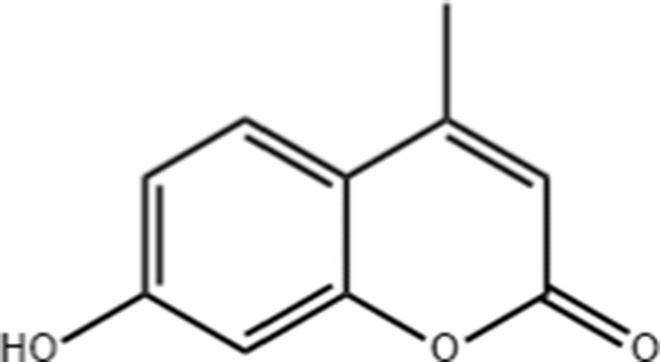	0.55

#### 2.7.2 Target prediction of phenolic compounds

The putative targets of the 17 bioactive compounds were retrieved from the PubChem database.^2^ The SMILES notations of these compounds were submitted to SwissTargetPrediction, and the Similarity Ensemble Approach (SEA)^3^ was used for target prediction. A Perl script was used to remove redundant targets and standardize the target lists. Gene symbols of the resulting targets were unified using UniProt database.

#### 2.7.3 Compilation of T2DM-related gene sets

T2DM-related genes were obtained from the GeneCards^4^ and OMIM^5^ databases. Redundant entries were removed, and the gene sets were merged and curated for further analyses.

#### 2.7.4 Screening of compound-disease common targets

The predicted targets of pitaya polyphenols and T2DM-related genes were intersected to identify common targets. A Venn diagram was generated using the R package “VennDiagram” to visualize the overlapping targets.

#### 2.7.5 Protein–protein interaction (PPI) network and visualization

A Perl script was used to integrate the compound–target and disease–target relationships in the network. The resulting compound–target–disease network was imported into Cytoscape for visualization and topological analysis. The PPI network of common targets was constructed using the STRING database with a confidence threshold of >0.9 and visualized in Cytoscape.

#### 2.7.6 Gene ontology (GO) and Kyoto encyclopedia of genes and genomes (KEGG) enrichment analysis

GO functional annotation and KEGG pathway enrichment analyses were performed on the common targets using the “clusterProfiler” R package. Significantly enriched terms (*p* < 0.05) were selected and visualized using bar plots and scatter plots. Key pathways related to glucose metabolism and T2DM were identified for further analysis.

### 2.8 Molecular docking

Molecular docking was performed using the SwissDock online tool. The three-dimensional structures of the target proteins (SRC and STAT3) were obtained from the RCSB PDB database.^6^ When multiple structures were available, the structure with the highest resolution was selected for further analysis. The protein structures were preprocessed using PyMOL to remove ligands and water molecules and then saved in the PDB format. The structures of the small-molecule ligands (naringenin, sinapic acid, and ferulic acid) were downloaded in SDF format from PubChem and converted to MOL2 format using OpenBabel. The prepared protein and ligand files were uploaded to SwissDock^7^ for molecular docking analysis.

### 2.9 Statistical analysis

The experiment was conducted using a completely randomized design. Data were analyzed using SPSS 20.0 (IBM Corp., Armonk, NY, United States) and are presented as mean ± standard deviation of three independent replicates. One-way analysis of variance (ANOVA) was performed to determine significant differences among the treatments, with statistical significance defined as *p* < 0.05. Data visualization and graph generation were performed using the Origin 2021 software (OriginLab Corp., Northampton, MA, United States).

## 3 Results and discussion

### 3.1 Microstructure of ozone-treated fresh-cut pitaya

The microstructural alterations in fresh-cut pitaya pulp, as visualized by SEM, provide direct evidence of the primary mechanism underlying the enhanced polyphenol extractability following ozone treatment. A comparative analysis of the control and ozone-treated groups on days 0 and 2 revealed significant differences in cell wall integrity and surface morphology.

**Figure 1:**
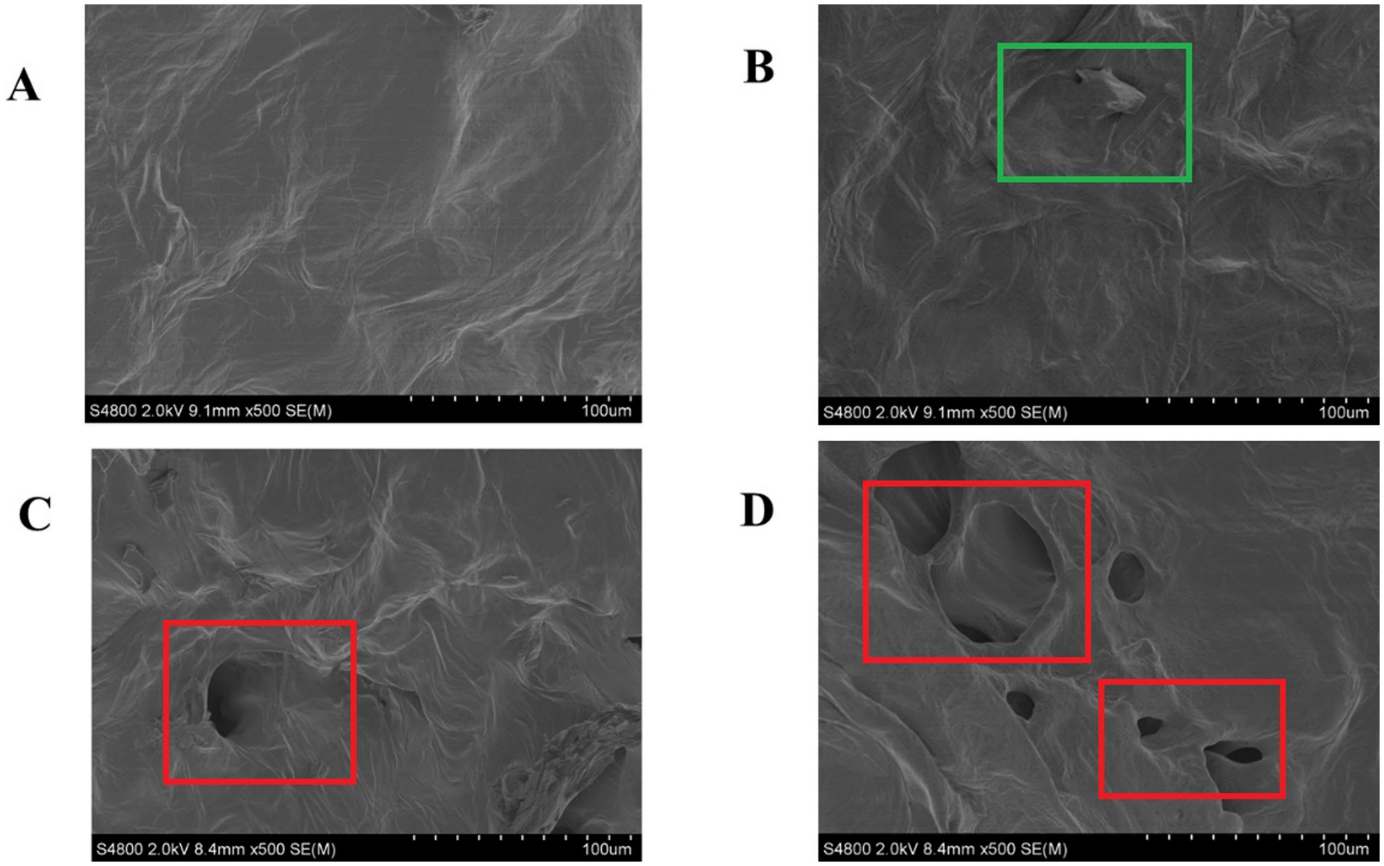
SEM images of the control group on day 0 **(A)** and day 2 **(B)** and ozone treatment on day 0 **(C)** and day 2 **(D)**.

As shown in Figures 1A,B, the cellular structure of the control samples remained relatively intact throughout the 2-day storage period. The cells were plump with smooth surfaces and the cell walls were tightly connected, forming a continuous non-porous matrix. This dense structure inherently limits the diffusion and release of intracellular components, including bound polyphenols.

In stark contrast, the microstructures of the ozone-treated samples exhibited profound changes immediately after treatment (Figure 1C). The cell surfaces appeared eroded and rough, and visible pores and microfissures were formed. This disruptive effect became even more pronounced after 2 days of storage (Figure 1D), showing extensive cell wall disintegration, collapsed cellular regions, and a notably more porous and fragmented network.

To objectively quantify these morphological changes, SEM micrographs were analyzed using the ImageJ software. The data clearly demonstrated that ozone treatment significantly (*p* < 0.05) increased the porosity and average pore size of pitaya tissue. The porosity of the ozone-treated group on day 0 (12.7%) was approximately 6 times greater than that of the control group on day 0 (2.1%). This difference increased as storage progressed.

Our findings align with studies on other fruits, where non-thermal treatments, such as ultrasound and pulsed electric fields, have been shown to mechanically disrupt cells and enhance polyphenol yield (19, 20). However, this study specifically elucidated the role of ozone as a chemical elicitor of microstructural porosity. This finding establishes that the enhancement of polyphenol extractability by ozone is not coincidental but is fundamentally driven by the targeted modifications of the physical architecture of plant tissue.

### 3.2 FTIR analysis of ozone-treated fresh-cut pitaya

**Figure 2:**
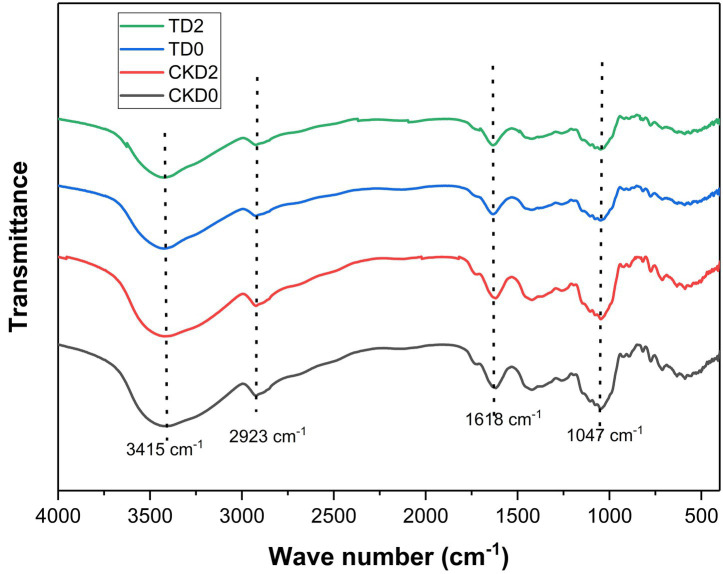
FTIR spectra of fresh-cut pitaya fruit in ozone treated group and control group.

Fourier-transform infrared (FTIR) spectroscopy was used to investigate the chemical alterations in the cell wall matrix of ozone-treated fresh-cut pitaya induced by ozone treatment. The comparative spectra of the control and ozone-treated groups (Figure 2) revealed consistent band profiles; however, notable changes were observed in transmittance intensity and subtle peak shifts, indicating significant modifications in the molecular structures of key components.

The broad, intense band centered at approximately 3415.81 cm^−1^ is attributed to O-H stretching vibrations, primarily from carbohydrates and water (21). The increased transmittance of the ozone-treated sample suggests a decrease in hydrogen bonding, which is likely due to the breakdown of the hydrated polysaccharide network in the cell wall. This is a direct consequence of the oxidative cleavage of the polymeric chains, which reduces the number of available hydroxyl groups for the intermolecular bonding.

A critical change was observed in the region between 1800 and 1,500 cm^−1^. The peak at approximately 1618.59 cm^−1^, assigned to the C=O stretching of esterified pectins (carboxylate groups) and possibly the aromatic C=C in some phenolics, showed altered intensity (22). The oxidative power of ozone is known to attack glycosidic linkages and break down pectin chains, leading to de-esterification and the formation of smaller and more soluble fragments. This degradation of the pectin matrix was directly correlated with the microstructural disintegration observed via SEM and facilitated the release of bound polyphenols. Similar ozone-induced depolymerization of pectin has been reported in other fruits, including apples and tomatoes.

The most pronounced changes occurred in the “fingerprint region” below 1,200 cm^−1^. The strong band at 1047.47 cm^−1^ is characteristic of C-O-C and C-O-H stretching vibrations in cellulose, hemicellulose, and glycosidic linkages (23). The significant increase in transmittance at this wavelength in the ozone-treated group strongly indicates the scission of these bonds. Ozone actively oxidizes the pyranose rings in cellulose and hemicellulose, leading to chain cleavage and a decrease in the overall polysaccharide content of the material. This results in a less dense and more fragmented cell wall, which mechanically explains the improved polyphenol extractability. The weakening of the 1,047 cm^−1^ band aligns with the findings of Dos Santos et al. (24), who observed analogous FTIR changes in ozone-treated plant tissues and linked them to the enhanced bioaccessibility of bioactives.

Furthermore, the band near 2,923 cm^−1^, corresponding to the C-H stretching in the CH₂ and CH₃ groups of carbohydrates, also showed increased transmittance. This further supports the premise of carbohydrate depolymerization, as the breakdown of polymer chains reduces the density of aliphatic C-H bonds.

In conclusion, the FTIR spectral data provide direct chemical evidence that ozone treatment induces the oxidative degradation of key structural polysaccharides—pectin, cellulose, and hemicellulose—in the cell wall of fresh-cut pitaya. This chemical breakdown, which manifested as the rupture of glycosidic linkages and weakening of the hydrogen-bonded network, was the fundamental mechanism behind the observed microstructural porosity and subsequent enhancement of polyphenol extractability and bioaccessibility.

### 3.3 Effect of ozone on phenolic content and antioxidant activity

#### 3.3.1 Phenolic content

**Figure 3:**
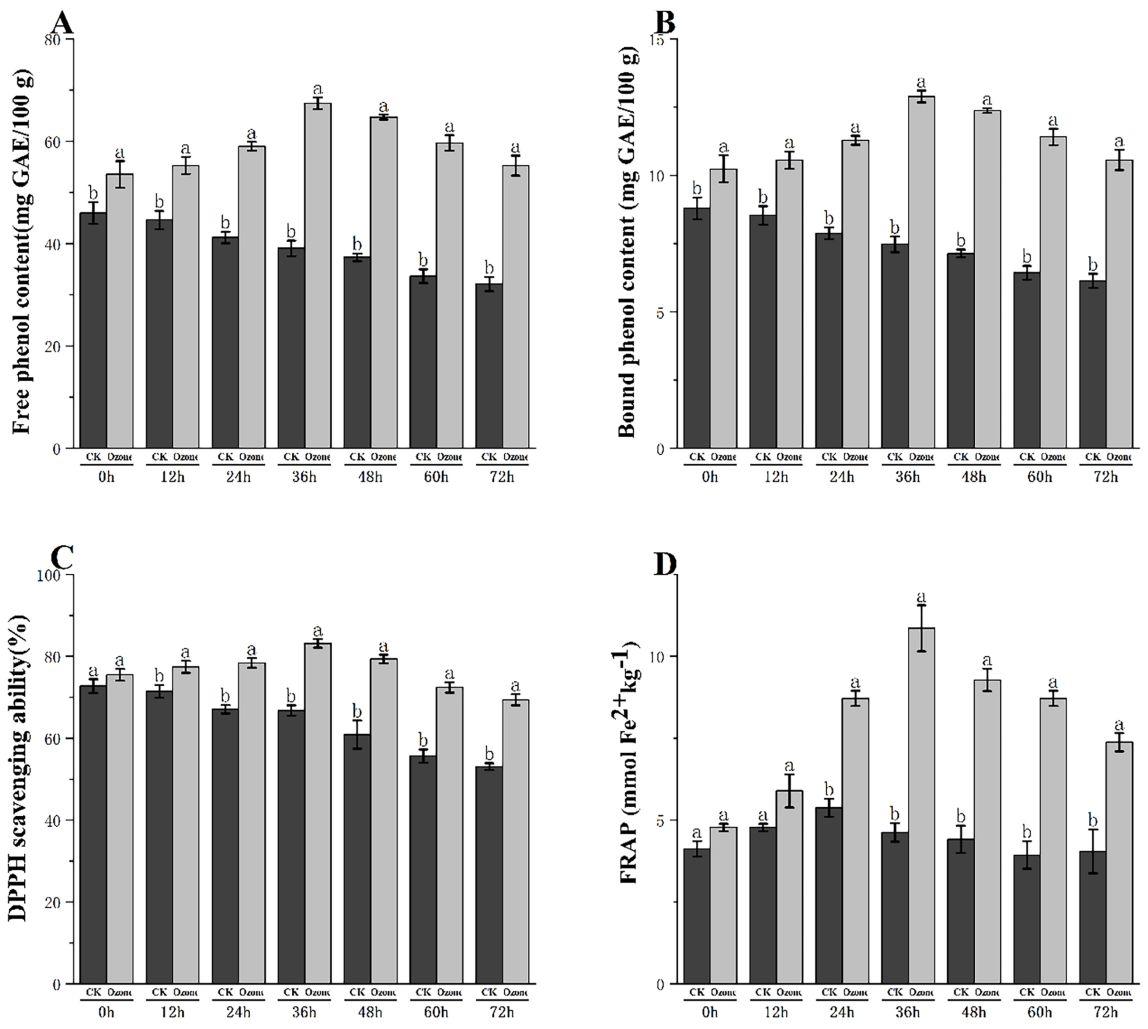
Free phenol content **(A)**, bound phenol content **(B)**, DPPH scavenging capacity **(C)** and FRAP **(D)** of fresh-cut Pitaya fruit in ozone treated group and control group.

The effect of ozone treatment on the phenolic compound profile and resultant antioxidant capacity of fresh-cut pitaya is shown in Figure 3. Ozone treatment elicited a significant and dynamic response in both free and bound phenolic fractions. Although the content of both phenolic forms gradually declined in the control group during storage, the ozone-treated fruits exhibited a marked increase, reaching a peak at 36 h (Figures 3A,B).

In the control group (CK), the free phenolic content decreased gradually over 72 h, from 45.2 mg GAE/100 g at 0 h to 32.1 mg GAE/100 g at 72 h. In contrast, the ozone-treated group showed a significant increase, peaking at 36 h (68.5 mg GAE/100 g) and 48 h (65.3 mg GAE/100 g), and remained substantially higher than that of the control throughout storage.

The bound phenolic content of the control group decreased slightly from 8.2 mg GAE/100 g (0 h) to 6.8 mg GAE/100 g (72 h). Meanwhile, the ozone-treated group exhibited consistently higher levels of bound phenolics, peaking at 36 h (12.3 mg GAE/100 g) and maintaining elevated levels during storage.

These results confirmed that ozone treatment enhanced the extractability of both free and bound phenolics and delayed their degradation during storage. The increase in bound phenolics was not due to *de novo* synthesis but rather to the release of phenolics from cell wall conjugates, as supported by our SEM (cell wall porosity/disruption) and FTIR (polysaccharide C-O-C bond degradation) analyses.

#### 3.3.2 Antioxidant activity

The DPPH scavenging ability of the control group decreased from 75.2% (0 h) to 52.8% (72 h). In contrast, the ozone-treated group showed a remarkable increase, peaking at 36 h (85.1%) and 48 h (82.3%) and remaining significantly higher than that of the control throughout the storage period (Figure 3C).

The FRAP value of the control group decreased from 3.2 μmol Fe^2+^/g (0 h) to 2.1 μmol Fe^2^/g (72 h). The ozone-treated group peaked at 36 h (10.5 μmol Fe^2+^/g) and maintained higher values during storage, consistent with the trend of phenolic content (Figure 3D).

Our results are consistent with those of other ozone-treated fruits, such as blueberries and mangoes, in which a similar elicitation of phenolic metabolism and enhancement of antioxidant activity have been documented.

Ozone treatment significantly improved the content of free and bound phenolics in fresh-cut pitaya by disrupting cell wall structures, thereby enhancing the antioxidant activity. This effect was sustained for over 72 h of storage, positioning ozone treatment as a promising technology for preserving and enhancing the nutritional quality of fresh-cut pitaya.

### 3.4 *In vitro* hypoglycemic activity

**Figure 4:**
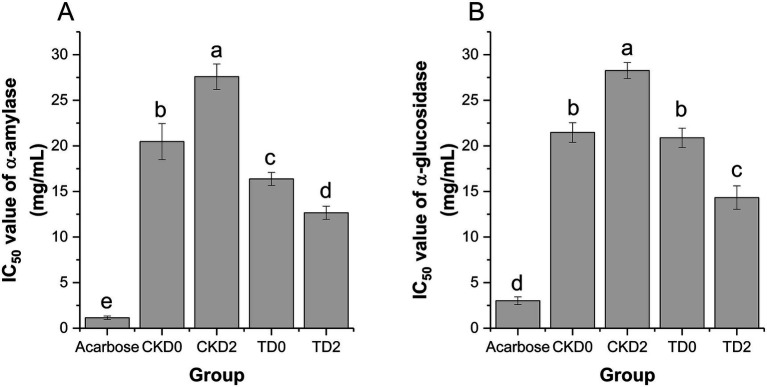
Inhibition rates of α-amylase **(A)** and α-glucosidase **(B)** in fresh-cut pitaya fruit on day 0 and day 2 in control group and ozone treatment group.

The half-maximal inhibitory concentration (IC₅₀) was used to evaluate the inhibitory activity of fresh-cut pitaya extracts against carbohydrate-hydrolyzing enzymes (*α*-amylase and α-glucosidase), with a lower IC₅₀ value indicates stronger enzyme inhibition. Acarbose, a commercial antidiabetic drug, was used as the positive control in this study (see Figure 4).

Acarbose exhibited the strongest *α*-amylase inhibition (IC₅₀ ≈ 0.8 mg/mL), serving as a benchmark for potent enzyme inhibition. In the control groups, CKD2 (control, stored for 2 days) showed a significantly higher IC₅₀ value (27.59 ± 1.40 mg/mL) than CKD0 (control, stored for 0 days) (20.47 ± 1.97 mg/mL), indicating that storage alone reduced *α*-amylase inhibitory activity (Figure 4A). In contrast, the ozone-treated groups demonstrated superior inhibition: TD0 (ozone, stored for 0 days) had an IC₅₀ of 16.37 ± 0.72 mg/mL, and TD2 (ozone, stored for 2 days) further decreased to 12.66 ± 0.72 mg/mL. Both values were significantly lower than those of their respective control counterparts (CKD0 and CKD2), confirming that ozone treatment enhanced *α*-amylase activity inhibition.

Acarbose also exhibited the strongest α-glucosidase inhibition (IC₅₀ ≈ 2.5 mg/mL). In the control groups, CKD2 (28.21 ± 1.53 mg/mL) had a higher IC₅₀ than CKD0 (21.35 ± 1.22 mg/mL), reflecting a diminished *α*-glucosidase inhibitory capacity after storage (Figure 4A). The ozone-treated groups exhibited robust inhibition: TD0 had an IC₅₀ of 20.86 ± 1.05 mg/mL, and TD2 decreased to 14.52 ± 0.98 mg/mL, both significantly lower than those of CKD0 and CKD2, respectively.

These findings are consistent with those of previous studies on phenol-rich foods. For example, it was reported that ozone treatment enhanced α-glucosidase inhibition in blueberry extracts by increasing free phenolic bioaccessibility (25), which is consistent with our observations in pitaya. Additionally, the structural diversity of polyphenols (e.g., number and position of hydroxyl groups) contributes to their enzyme-inhibitory efficacy. Thus, the ozone-induced release of specific polyphenolic compounds in pitaya may further explain the potent hypoglycemic activity observed.

In summary, ozone treatment improved the *in vitro* hypoglycemic potential of fresh-cut pitaya by strengthening the inhibition against α-amylase and α-glucosidase. This effect is primarily mediated by increased polyphenol bioaccessibility and preserved structural integrity during storage, positioning ozone-treated fresh-cut pitaya as a promising functional food for managing postprandial blood glucose levels.

### 3.5 Bioaccessibility of polyphenols after *in vitro* digestion

The stability and release of polyphenols throughout the simulated gastrointestinal tract were quantitatively assessed to determine their bioaccessibility, a critical factor in predicting their potential health benefits. As detailed in Table 2, ozone treatment markedly enhanced the polyphenol content at every stage of digestion and significantly improved the overall bioaccessibility.

**Table 2:** Polyphenol contents and bioaccessibility of polyphenols in fresh-cut pitaya.

Substance	Undigested		Oral digestion		Gastric digestion		Intestinal digestion		Bioaccessibility	
	mg GAE/100 g								%	
	CK	Ozone	CK	Ozone	CK	Ozone	CK	Ozone	CK	Ozone
Fresh-cut Pitaya powder	48.07 ± 1.50a	76.40 ± 1.14a	43.26 ± 1.35b	68.76 ± 1.03b	33.65 ± 1.05c	59.10 ± 1.75c	19.46 ± 1.91d	36.79 ± 4.03d	40	48
Free polyphenols	39.08 ± 1.50a	67.41 ± 1.14a	34.90 ± 0.98b	60.22 ± 0.75b	27.61 ± 0.70c	49.87 ± 0.94c	16.88 ± 3.81d	33.42 ± 3.64d	43	50
Bound polyphenols	7.47 ± 0.29a	12.89 ± 0.22a	6.36 ± 0.63b	10.31 ± 0.18b	5.28 ± 0.13c	9.54 ± 0.18c	3.10 ± 0.57d	5.50 ± 0.50d	41	43

The initial total phenolic content in the undigested pitaya powder was 48.07 mg GAE/100 g in the control (CK) and 76.40 mg GAE/100 g in the ozone-treated group, confirming the enhanced extractability reported previously. This superior starting point was maintained throughout the digestive process. After the intestinal phase, the final concentrations of bioaccessible polyphenols were 19.46 mg GAE/100 g for CK and 36.79 mg GAE/100 g for the ozone group. Consequently, the overall bioaccessibility of the total polyphenols increased from 40% in the control to 48% in the ozone-treated samples.

A more detailed analysis of the phenolic forms revealed that both free and bound polyphenols benefited from this treatment. The bioaccessibility of free polyphenols increased from 43% (CK) to 50% (Ozone), whereas that of bound polyphenols increased from 41 to 43%. Notably, the free polyphenols consistently exhibited higher bioaccessibility than the bound fraction in both groups, and the absolute amount of bioaccessible free phenolics in the ozone group (33.42 mg GAE/100 g) after digestion was nearly double that of the control (16.88 mg GAE/100 g).

A progressive decline in polyphenol content from the oral to intestinal phase is expected, as the harsh conditions of the gut (e.g., alkaline pH, digestive enzymes, and bile salts) can degrade sensitive phenolic compounds. However, the ozone-treated samples demonstrated not only higher initial levels but also greater resilience during this process.

The primary reason for the enhanced bioaccessibility is directly linked to the ozone-induced microstructural modifications. As demonstrated by SEM and FTIR, the breakdown of the cell wall matrix created a more porous structure, which likely allowed for a more efficient and complete release of polyphenols during digestion. Once liberated from the dense cellular matrix, these compounds are more readily available to enter the bioaccessible fraction. Furthermore, oxidative treatment may have broken down some larger, complex polyphenol-polyaccharide conjugates into smaller, more stable molecules that are less susceptible to degradation in the gastrointestinal environment.

The higher bioaccessibility of free polyphenols aligns with their chemical nature; as they are less tightly bound to the cell wall, they are more easily solubilized and released into digestive chyme. The modest but significant improvement in the bioaccessibility of bound polyphenols is particularly noteworthy. This suggests that ozone treatment partially predigests the cell wall, facilitating the release of this otherwise recalcitrant fraction, which is typically excluded from the bioaccessible pool (26).

Our findings are consistent with the growing body of evidence that physical and chemical pretreatments can enhance the bioaccessibility of bioactive plant compounds. Rinaldi, M., et al. (27) reported that ohmic heating, another cell-disrupting technology, improved the bioaccessibility of phenolic compounds in pineapple waste. The present study established ozone as an effective technology for fresh-cut pitaya.

In conclusion, by structurally priming plant tissues, ozone treatment ensured that a greater proportion of the extracted polyphenols survived the digestive process and remained available for potential intestinal absorption, thereby significantly increasing the functional food value of the plant.

### 3.6 *In silico* prediction of hypoglycemic potential

**Figure 5:**
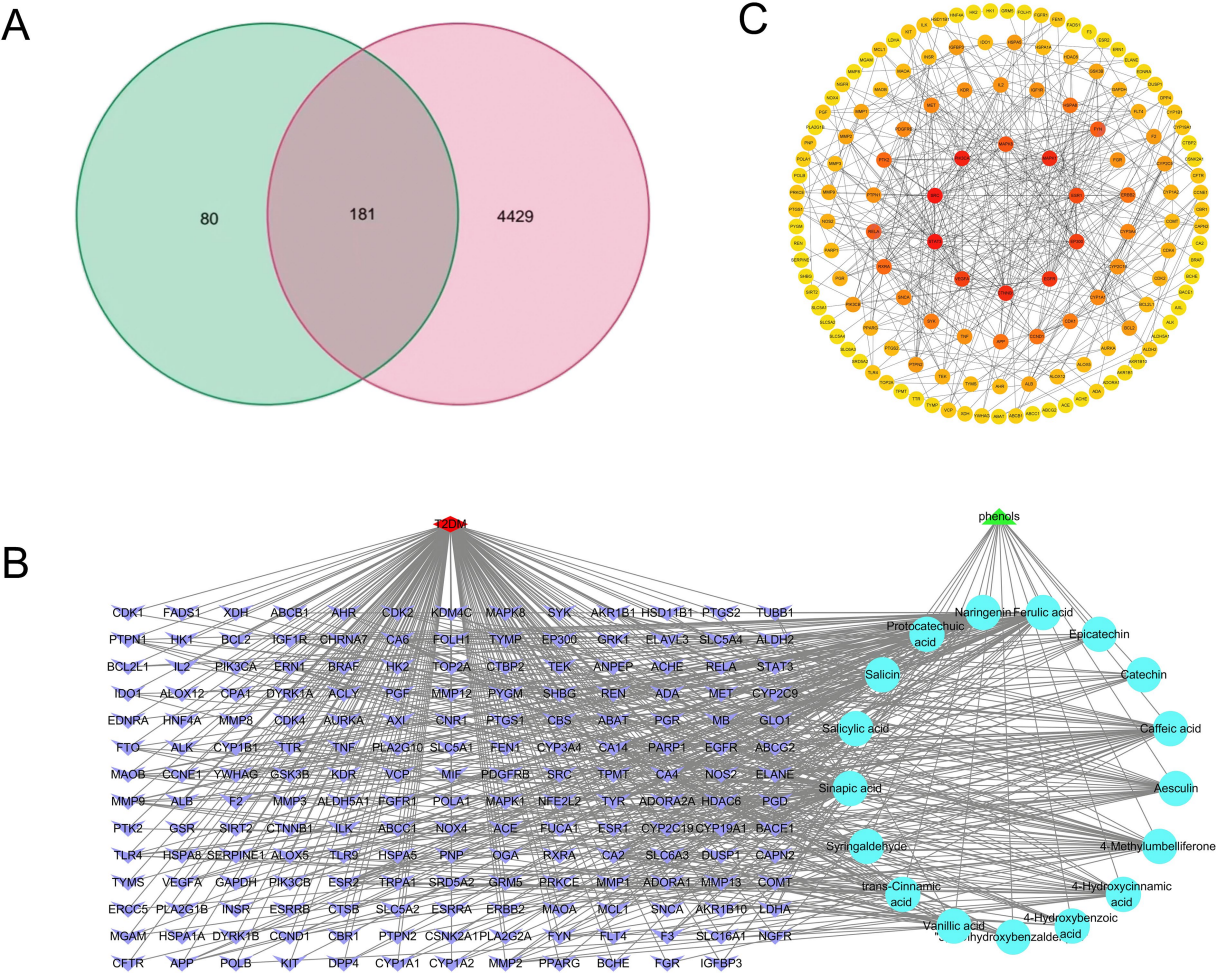
Venn diagram of polyphenols and disease targets **(A)**; fresh-cut pitaya polyphenols - components - common targets **(B)**; mutual mapping of common target PPI **(C)**.

To elucidate the potential molecular mechanisms underlying the observed *in vitro* hypoglycemic activity, an integrated network pharmacology approach was used. The analysis identified 181 common targets shared between the identified pitaya polyphenols and type 2 diabetes mellitus (T2DM) (Figure 5A), suggesting a multi-target modulatory potential. The compound-target network (Figure 5B) highlighted the key bioactive compounds, including naringenin, ferulic acid, and sinapic acid, as the central players that interact with numerous targets. Subsequent protein–protein interaction (PPI) network analysis of these common targets (Figure 5C) identified several core hub genes, such as SRC, STAT3, MAPK1, and EGFR, which are known to be critically involved in insulin signaling and inflammation pathways.

**Figure 6:**
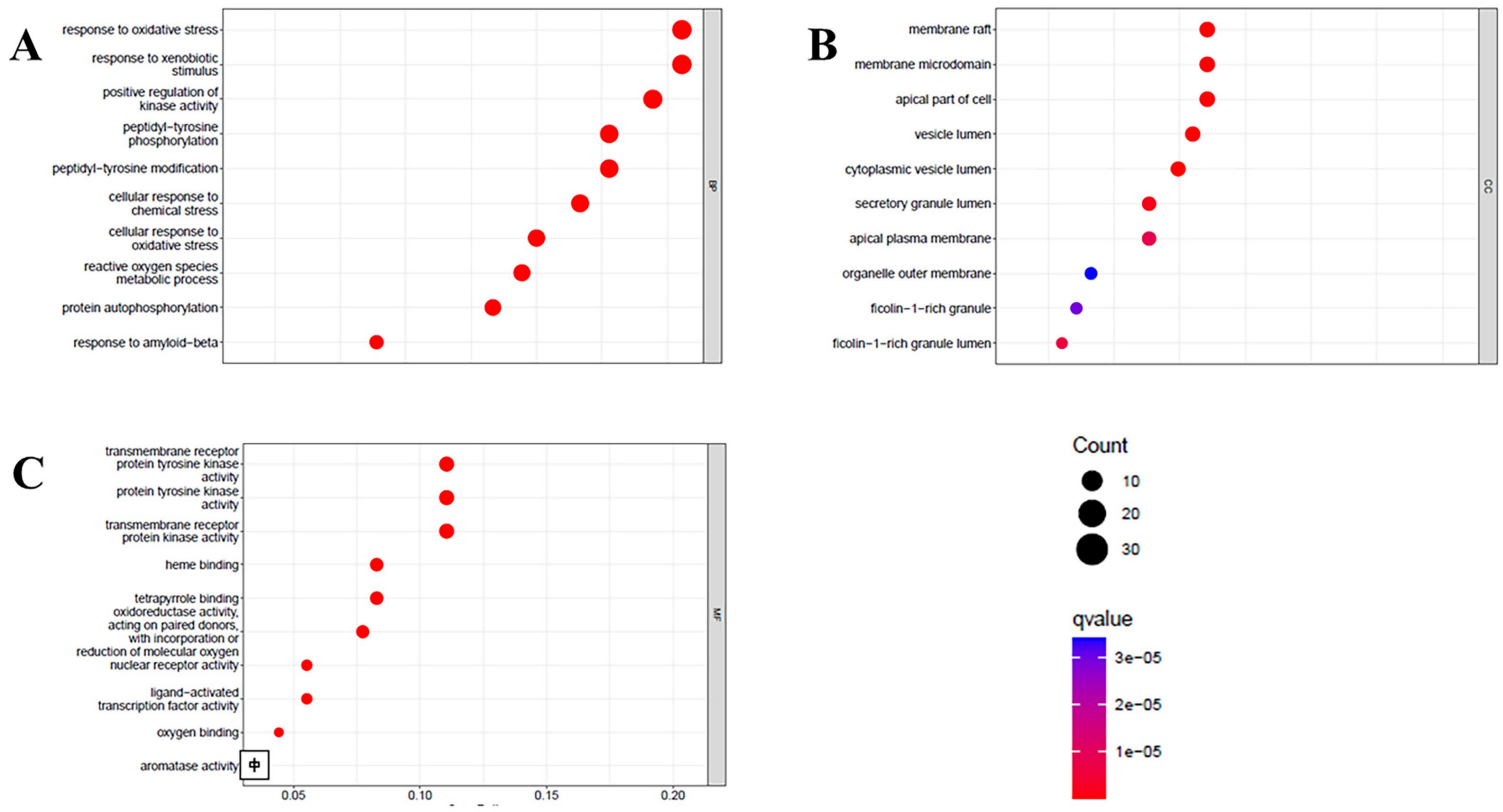
Biological processes **(A)**, cell component **(B)**, and molecular function **(C)** of GO enrichment analysis.

Gene Ontology (GO) enrichment analysis provided insight into the biological functions of these common targets (Figure 6). The results were highly significant, revealing a strong association between the response to oxidative stress and reactive oxygen species metabolic processes (Biological Process), underscoring the potential of these polyphenols to ameliorate oxidative damage, a key pathological factor in insulin resistance. Notably, the top Molecular Functions were transmembrane receptor protein tyrosine kinase activity and protein tyrosine kinase activity, which are directly implicated in the initial steps of insulin receptor signaling. This suggests that polyphenols potentiate insulin activity by interacting with and modulating these kinases.

**Figure 7:**
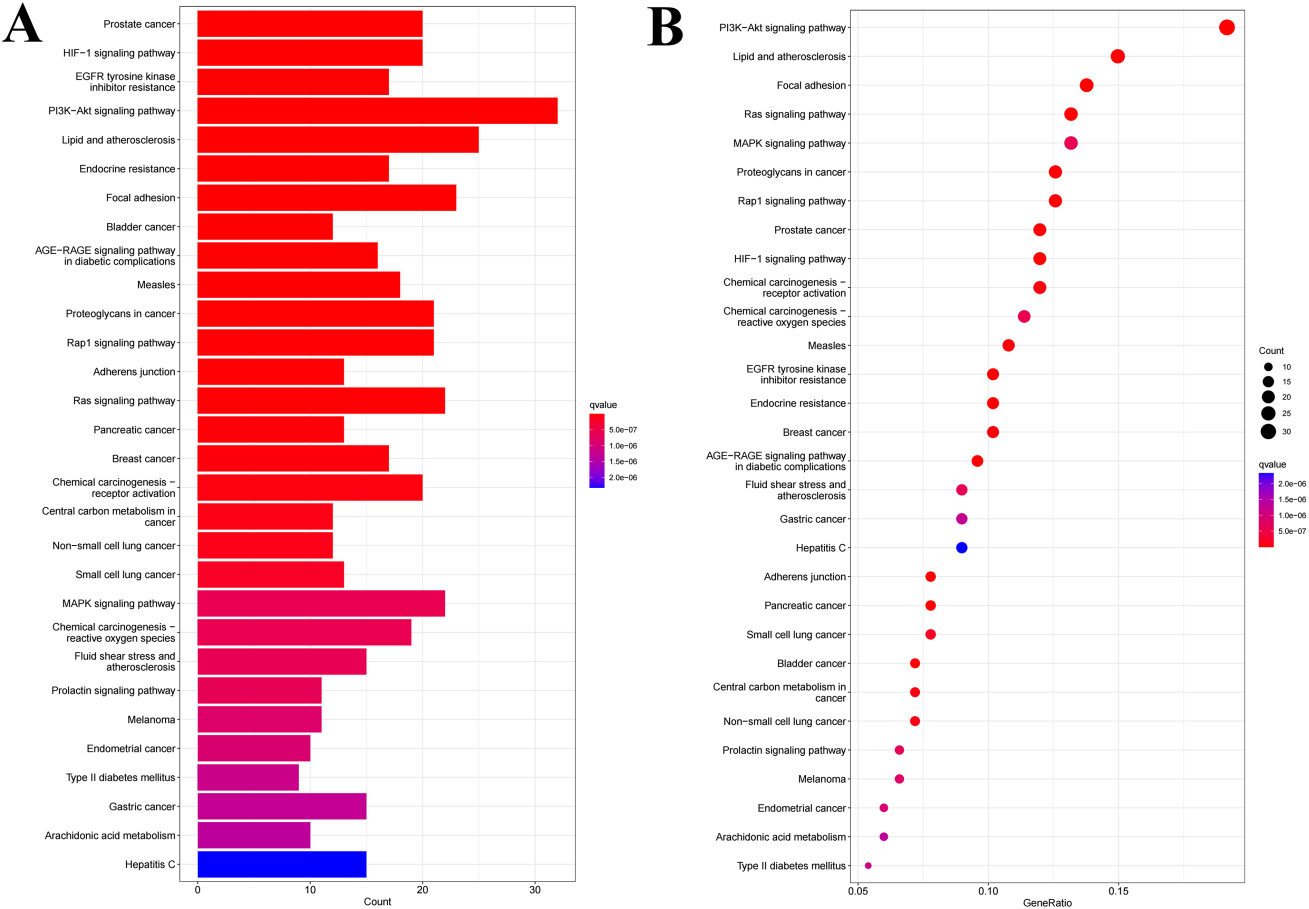
Column diagram **(A)** and bubble diagram **(B)** of KEGG enrichment analysis.

Kyoto Encyclopedia of Genes and Genomes (KEGG) pathway analysis confirmed the hypothesized mechanisms (Figure 7). The most significantly enriched pathways in diabetic complications were the PI3K-Akt signaling pathway, endocrine resistance, and AGE-RAGE signaling pathway. The enrichment of the PI3K-Akt pathway is of paramount importance because it is the primary intracellular pathway that transduces the metabolic effects of insulin, including glucose uptake and glycogen synthesis. The hub genes identified in the PPI network, such as SRC and STAT3, are known to be upstream regulators and effectors of this pathway. Furthermore, the enrichment of the AGE-RAGE pathway suggests an additional protective role, potentially inhibiting the formation of advanced glycation end products and associated chronic inflammation that exacerbates diabetes.

### 3.7 Molecular docking

**Figure 8:**
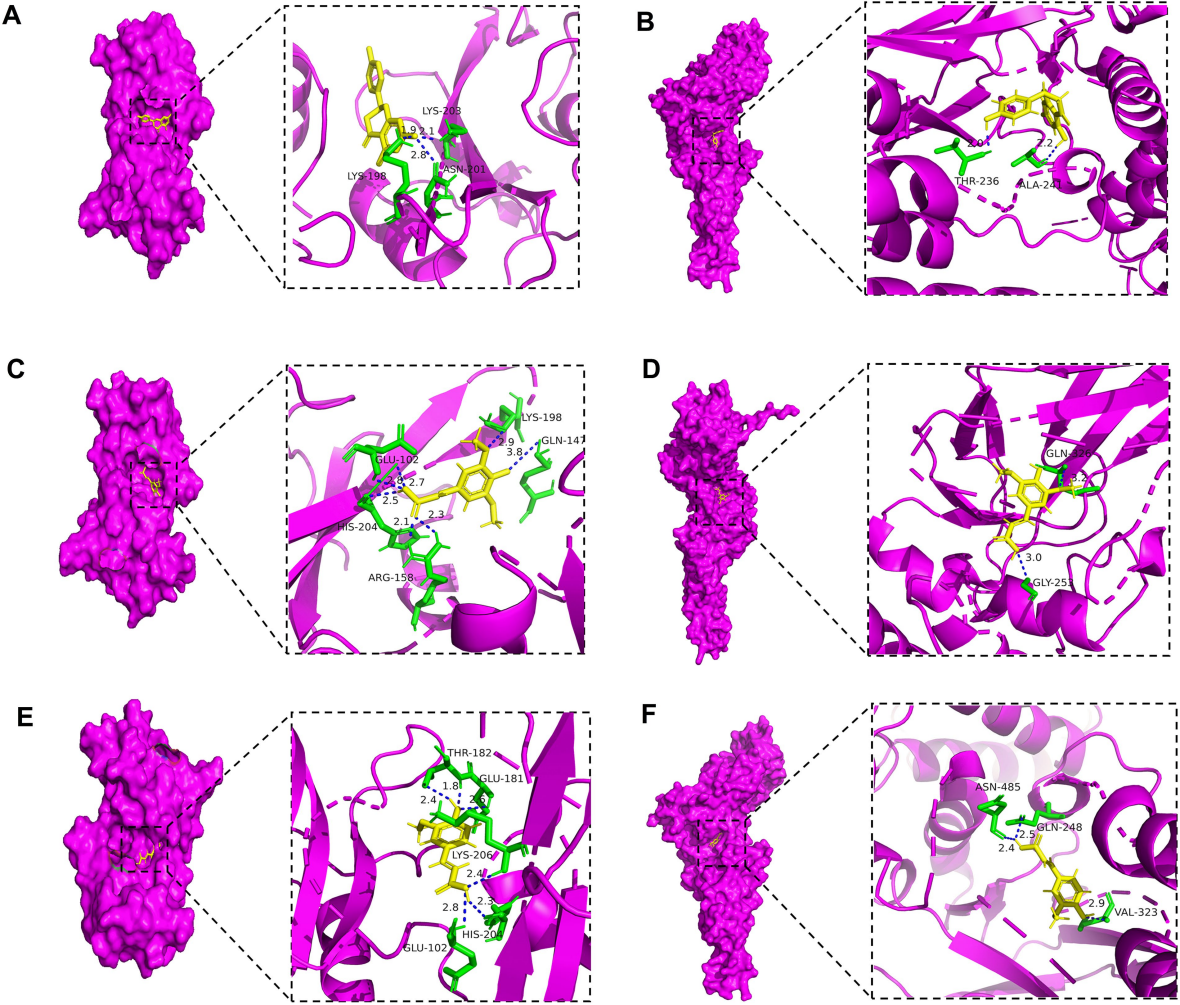
Results of molecular docking between active ingredients and disease targets.

The *in silico* molecular docking results (Figure 8 and Table 3) provided direct binding evidence, showing that key compounds such as naringenin and ferulic acid exhibited strong binding affinities (energy < −5.0 kcal/mol) with the hub targets SRC and STAT3. This computationally predicted interaction provides a structural basis for the proposed modulation of the PI3K-Akt pathway.

**Table 3:** Binding energies of three key active ingredients and two key targets (kcal/mol).

Key active ingredients	Key targets	
	SRC	STAT3
Naringenin	−7.5	−5.6
Sinapic acid	−6.1	−6.0
Ferulic acid	−6.1	−6.7

In summary, *in silico* analyses predicted that the hypoglycemic effect of ozone-treated pitaya polyphenols is not a singular event but a synergistic outcome of multi-target regulation. The proposed mechanism involves (1) the direct inhibition of carbohydrate-hydrolyzing enzymes (*α*-glucosidase/α-amylase), as confirmed *in vitro*; it also involves (2) the potential activation of the insulin-sensitive PI3K-Akt signaling pathway and mitigation of diabetic complications via interaction with core targets such as SRC and STAT3, while reducing oxidative stress. This systems-level explanation significantly enhances the scientific depth of our findings and provides a robust theoretical foundation for future *in vivo* studies.

## 4 Conclusion

In summary, this study demonstrated that ozone treatment is an effective postharvest strategy for enhancing the functional value of fresh-cut pitayas. The key mechanism was identified as the disruption of the fruit cell wall microstructure, which facilitated the release of bound polyphenols. This improved extractability significantly increased polyphenol bioaccessibility after *in vitro* digestion. Consequently, the ozone-treated samples exhibited markedly stronger *in vitro* hypoglycemic activity, as evidenced by their superior inhibition of α-amylase and α-glucosidase enzymes.

Furthermore, our integrated approach, which combines *in silico* and *in vitro* analyses, provides deeper mechanistic insights. Molecular docking identified naringin, erucic acid, ferulic acid, and caffeic acid as key bioactive compounds, with SRC, STAT3, and PIK3CA emerging as potential targets. This suggests that the hypoglycemic effect of ozone-treated pitaya polyphenols is not only a result of enzyme inhibition but may also involve modulation of the PI3K-AKT signaling pathway, indicating a potential multi-target mechanism for regulating glucose metabolism.

Based on these findings, future research should prioritize *in vivo* studies to validate the physiological hypoglycemic effects of these compounds and further elucidate the molecular mechanisms underlying the modulation of the PI3K-AKT pathway. This study demonstrates the potential of ozone treatment as a clean-label technology for producing fresh-cut fruits with enhanced health benefits.

## Data Availability

The original contributions presented in the study are included in the article/supplementary material, further inquiries can be directed to the corresponding author.

## Footnotes

1 http://www.swissadme.ch/.

2 https://pubchem.ncbi.nlm.nih.gov/.

3 https://sea.bkslab.org/.

4 https://www.genecards.org/.

5 https://www.omim.org/.

6 https://www.rcsb.org/.

7 http://www.swissdock.ch/.
